# The effect of Traumeel LT ad us. vet. on the perioperative inflammatory response after castration of stallions: a prospective, randomized, double-blinded study

**DOI:** 10.3389/fvets.2024.1342345

**Published:** 2024-10-02

**Authors:** Julia Laves, Melanie Wergin, Natali Bauer, Simon Franz Müller, Klaus Failing, Kathrin Büttner, Alina Hagen, Michaela Melzer, Michael Röcken

**Affiliations:** ^1^Equine Clinic (Surgery and Orthopedics), Justus-Liebig-University, Giessen, Germany; ^2^Biologische Heilmittel Heel GmbH, Baden-Baden, Germany; ^3^Department of Veterinary Clinical Sciences, Clinical Pathology and Clinical Pathophysiology, Justus-Liebig-University Giessen, Giessen, Germany; ^4^Laboklin GmbH & Co. KG, Bad Kissingen, Germany; ^5^Unit for Biomathematics and Data Processing, Justus-Liebig-University, Giessen, Germany

**Keywords:** acute phase reaction, inflammation, anti-inflammatory drug, inflammation resolution, castration, Traumeel, wound healing, serum-amyloid A

## Abstract

**Introduction:**

Stallion castration is a standard procedure with a risk of post-surgical complications. Castration induces an acute phase response (APR). Serum Amyloid-A (SAA) is a well-studied major acute phase protein (APP), that has been shown to be a good marker for the development of post-surgical complications. The current gold standard for reducing the APR after castration is Flunixin-Meglumin, which is a non-steroidal anti-inflammatory drug (NSAID) inhibiting COX1/2. In contrast, Traumeel LT ad us. vet. can modulate the APR by induction of the inflammation resolution. The aim of this study was to compare the effect of Flunixin-Meglumin and Traumeel LT ad us. vet. on the acute phase response.

**Material and methods:**

A total of 60 stallions were recruited and 54 stallions entered the study with 27 stallions in each treatment group. The stallions were treated pre- and postoperatively with either Flunixin-Meglumin (FL) or with Traumeel LT ad us. vet. (TR). Blood was taken before and 24 h, 48 h and 72 h after castration. The following main parameters were assessed: SAA, fibrinogen, iron, white blood cells, neutrophils, Interleukin1ß, and cortisol. Wound healing and pain were assessed at 8 time points.

**Results:**

The main variable SAA was increased after surgery reaching a mean value of 122 µg/ml in the FL group and a mean SAA of 226 µg/ml in the TR group 48 h after surgery, reaching a significant difference only at the 24 h timepoint (*p* = 0.03). All stallions had the highest pain summary score 8 hours after surgery, with decreasing values thereafter. The pain scores were not statistically different at any time point. In the FL group five stallions developed a suture dehiscence compared to only one stallion in the TR group (*p* = 0.001).

**Discussion:**

Within the limitations of this study, Traumeel LT ad us. vet. seems to have proresolving effects on the inflammation induced by surgery making it a valuable treatment to reduce the APR induced by castration. Due to its different mode of action, Traumeel LT ad us. vet. might be an alternative treatment option if gastrointestinal side effects or renal side effects of NSAIDs should be avoided. Further studies are needed combining Traumeel LT ad us. vet. and Flunixin.

## Introduction

Knowing that every surgical procedure is causing a soft tissue trauma, and that tissue trauma leads to a well-orchestrated inflammatory reaction, it is of utmost importance to understand the response to wounding of the body to assure proper wound healing (1, 2).

The inflammatory reaction is necessary to allow the body to adapt to surgical trauma and to promote healing. An exuberant inflammatory response is predictive for complications. To assess the severeness of the inflammatory reaction, several biomarkers have been proven useful in horses. The systemic measurable reaction is named acute phase response (APR) (3). The systemic APR is a complex set of inflammatory reactions induced by tissue damage or infection. The APR is initiated by the release of several acute phase proteins (APP) (4). One important group of APPs are cytokines, such as interleukin (IL) 1ß, IL6, and tumor necrosis factor *α* (TNF-α). These cytokines can induce the production of Serum Amyloid A (SAA) in the liver (5, 6). Haptoglobin and fibrinogen are also significantly increased after tissue damage and are classified as moderate APR. (7–9).

The most widely used APP in equine medicine is SAA (6, 10–12). Its production is initiated by IL 1ß and IL 6 in the liver within 6 h after the insult leading to an increase up to 1,000-fold to defend inflammation and to promote tissue healing (5, 13). SAA has a short half-life of 30–120 min (14, 15) making it a valuable monitor for the course of inflammation. A pronounced increase in SAA correlates to an increased risk of postoperative complications after castration of stallions (3, 6, 16, 17). Stallion castration is a standard procedure in equine medicine with a risk of post-surgical complications, such as oedema, seroma, delayed wound healing with suture dehiscence, and infections as the main complication risks (18–21).

Surgical procedures are known to cause considerable pain. For animal welfare reasons, sufficient pain control post-surgery is mandatory (22–24). Non-steroidal anti-inflammatory drugs (NSAID) are routinely used for their analgesic and anti-inflammatory effect. Flunixin-Meglumin (Phlogoxin, SelectaVet GmbH, Germany) is a frequently used drug to treat mild visceral pain and to reduce the inflammatory reaction (25). Recent guidelines recommend treatment with NSAIDs, for example Flunixin-Meglumin before and up to 3 days after surgery to reduce postoperative pain and to limit the inflammatory reaction (26). NSAIDs are known to be more effective as analgesic, when inflammation is part of the pain process due to down regulation of IL1ß (27, 28). Besides the positive effects of the non-selective cyclooxygenase (COX) inhibitor, Flunixin-Meglumin also has considerable side effects, such as, but not limited to, gastrointestinal ulceration and impaired renal function (29).

Flunixin-Meglumin inhibits the synthesis of prostanoids such as prostaglandin-E2 (PGE2), prostacyclins, and thromboxanes by blocking both cyclooxygenase 1 (COX-1) and cyclooxygenase 2 (COX-2) enzymes (28). In contrast, Traumeel (registered for Human use, Heel GmbH, Germany) has been shown to regulate several pathways associated with the resolution of acute inflammation, including apoptosis, leukocyte migration, and angiogenesis in a murine wound healing model (2, 30). Traumeel has a positive impact on the synthesis of specialized pro-resolving mediators (SPMs) in human monocyte-derived macrophages and thereby Traumeel is enhancing efferocytosis of neutrophiles by macrophages and SPM production in a zymosan-induced mouse model (31). In previous double-blinded, randomized controlled trials, Traumeel has been shown to reduce pain, one of the hallmarks of acute inflammation, after musculoskeletal injury in human patients (32, 33). In addition, Traumeel inhibits IL1ß and TNF-*α* production by resting and activated immune cells *in vitro* (2).

The present prospective, double-blinded, randomized clinical trial obtained data on perioperative clinical inflammatory symptoms (internal body temperature, wound assessment), pain (frequent pain scoring), and changes in blood markers reflecting APR and pain-related stress reaction (SAA, fibrinogen, iron, white blood cells (WBC), neutrophils, IL1ß, and cortisol).

The aim of our study was to objectively assess the perioperative inflammatory response after castration of stallions comparing Traumeel LT ad us. vet. to the non-steroidal anti-inflammatory drug Flunixin-Meglumin, hypothesizing that Traumeel LT ad us. vet. would reduce SAA values comparable to Flunixin-Meglumin and Traumeel LT ad us. vet. would reduce wound healing complications.

## Materials and methods

This prospective, double blinded, randomized clinical study was approved by the ethical committee of the Giessen regional council in Hessen (Germany) with the number G 94/2019. Owners gave written informed consent for the inclusion of their stallions into the study.

### Animals

A total of 60 stallions were recruited for the study between March 2020 and September 2021. The inclusion criteria for the study were met by 57 clinically healthy stallions. Inclusion criteria were no treatment with NSAIDs or bioregulatory medications within the previous 4 weeks, an unremarkable clinical examination, testicles in the scrotum at least under sedation, a serum SAA <45 mg/mL, and hematology and clinical chemistry within normal limits. Stallions with a diagnosed acute or chronic kidney disease, a history of gastritis and/or gastric ulcers or vaccination within the last 10 days were excluded. The 57 stallions were randomized by a matched pair randomization. Randomization was done for the parameter age, weight, and baseline SAA. The matched-pair randomization was done by veterinarians not working at or being connected to the horse clinic. From the 57 stallions, 54 stallions had a match and were therefore included in the study leaving 27 stallions per group.

One day after arrival, an indwelling catheter was placed in an external jugular vein in all horses and blood samples were taken for hematology and clinical chemistry, including SAA.

### Treatment

The drugs were prepared by the veterinarians responsible for the matched pair randomization. The drugs were delivered to the clinic in opaque syringes to assure blinding (Supplementary Figure S1). Due to the different application routes either intravenous or subcutaneous and time points, horses in both groups received one verum dose and one placebo (NaCl 0.9%) at the indicated time points.

Horses in the Flunixin group were treated with Flunixin-Meglumin (Phlogoxin, SelectaVet GmbH) with 1.1 mg/kg BW i.v. every 24 h as recommended in the package leaflet. The medication was administered at three time points. The first dose was given approximately 15 min before anesthesia, then 24 and 48 h after castration. In this group each horse received NaCl 0.9% subcutaneous as placebo to assure the blinding of the study.

Horses in the Traumeel group (TR) received Traumeel LT ad us. vet. (registered for Veterinary use, Heel GmbH, Germany) as subcutaneous injections in an aqueous solution. Horses weighing <500 kg BW received 5 mL and horses ≥500 kg BW received 10 mL as recommended by the manufacturer. One ampoule with 5 mL contains the following ingredients: *Aconitum napellus* Dil. D4, 300 mg; *Arnica montana* Dil. D4, 500 mg, *Atropa bella-donna* Dil. D4, 500 mg; *Bellis perennis* Dil. D4, 250 mg; *Calendula officinalis* Dil. D4, 500 mg; *Matricaria recutita* Dil. D5, 500 mg; Echinacea Dil. D4, 125 mg; *Echinacea purpurea* e planta tota Dil. D4, 125 mg; *Hamamelis virginiana* Dil. D4, 50 mg; *Hypericum perforatum* Dil. D4, 150 mg; *Achillea millefolium* Dil. D5, 500 mg; *Symphytum officinale* Dil. D8, 500 mg; Hepar sulfuris Dil. D6 aquos., 500 mg; Mercurius solubilis Hahnemanni Dil. D8, 250 mg. The first dosage was administered approximately 15 min before anesthesia, then the TR group received Traumeel LT ad us. vet. every 12 h up to 48 h after castration. In this group each horse received NaCl i.v. as placebo to assure the blinding of the study.

Butorphanol (Butorgesic 10 mg/mL, CP-Pharma, Burgdorf) was chosen as rescue pain medication because it can be safely combined to Flunixin-Meglumin and Traumeel LT ad us. vet. thus, not breaking the blinding if Butorphanol had to be given. Butorphanol was allowed to be given twice every 6 h if the horse grimace scale was >4 and/or the Composite Pain Scale was >12.

### Castration

All stallions were castrated under general anesthesia in dorsal recumbency with an additional local anesthesia infiltrated in the testis toward the spermatic cord.

Food was withheld for 4–6 h but horses had access to water until premedicated. Premedication was given approximately 15 min before sedation with a combination of an α2-agonist (Xylazin: 0.8 mg/kg BW i.v., Xylavet® 20 mg/mL, CP-Pharma, Burgdorf, Germany) and an opioid (Butorphanol: 0.05 μg/kg BW i.v., Butorgesic® 10 mg/mL, CP-Pharma, Burgdorf, Germany).

General anesthesia was induced with ketamine 2.5 mg/kg BW i.v. (Anesketin, 100 mg/mL, Dechra Veterinary Products Deutschland GmbH, Aulendorf, Germany) and diazepam 0.05 mg/kg BW i.v. (Ziapam® 5 mg/mL, Dechra Veterinary Products Deutschland GmbH, Aulendorf, Germany).

After orotracheal intubation and positioning in dorsal recumbency, anesthesia was maintained with a balanced protocol with isoflurane (Isofluran CP® 1 mL/mL; CP-Pharma, Burgdorf, Germany) in 100% oxygen and continuous drip infusion with xylazine (0.3–0.8 mg/kg per hour i.v.) adjusted to maintain a stage III, plane 2 anesthesia depth. Respiratory and cardiovascular parameters, as well as arterial blood pressure and oxygen saturation were monitored throughout the surgical procedure. Arterial blood gas analysis (Cobas b® 123, Roche Diagnostics, Mannheim, Germany) was performed every 20 min during anesthesia. All horses received Ringer’s solution (5–10 mL/kg per hour i.v.) for supply of fluid and electrolytes. Dobutamine was infused at a rate of 0.25–1 μg/kg per min i.v. to maintain MAP (mean arterial pressure) > 60 mmHg.

Two experienced surgeons performed the castration following the same standard operating procedure. After aseptic preparation of the surgical field Lidocaine (Lidocainhydrochlorid 2%, 20 mg/mL, Bela-pharm GmbH & Co. KG, Germany) was injected at a dose of 0.4 mg/kg BW intratesticular to each testis (this dose corresponds to 10 mL per testis in a 500 kg horse) (34). After the injection of Lidocaine two skin incisions with a length of approximately 7 cm were made over the superficial inguinal ring. The underlying fascia was incised, and the tissue was prepared blunt until the testis in the vaginal tunic were exposed. After dissecting the musculus cremaster with the Ligasure™, the spermatic cord was crushed with the Sand’s emasculator and a double ligature was made around the spermatic cord (Safil®, USP 1, B. Braun Surgical, S.A. Rubi, Spain). After inspection for hemorrhage, the pedicle was released and the wound was primary closed in two layers with absorbable 2–0 USP monofilament suture using a horizontal-mattress suture for the fascia (Biosyn™, Covidien, Neustadt/Donau, Germany) and a horizontal-mattress suture for the intra-dermal and for the cutaneous incision (Novosyn®, USP 2–0, B. Braun Surgical, S.A. Rubi, Spain). All horses received soft food around 3 h after recovery and normal hay afterwards.

### Post operative care

Post operative care included physical examination, pain scoring and wound evaluations. On the day of castration, the horses were intensively monitored every 4 h (4, 8 and 12 h after surgery). Subsequent examinations were performed at 24, 36, 48 and 72 h after castration.

For wound evaluation incisional swelling, suture adaptation and wound secretion were scored for each suture separately. Incisional swelling was classified as no swelling, mild (<0.5 cm diameter), moderate (≥0.5–2 cm diameter), severe (>2–5 cm diameter) or intense (>5 cm diameter). Suture dehiscence was classified as no dehiscence, mild (up to 25%), moderate (25–50%), severe (>50–75%) or complete suture dehiscence. Secretion was described as serous, sanguinary, or purulent.

The first pain evaluation was performed the day before castration to establish the individual baseline value. For scoring, a modified Composite Pain Scale (CPS) (35) (Supplementary Table S2a) and the Horse Grimace Scale (HGS) (Supplementary Table S2b) (36) were used. For performing the pain score, the blinded examiner initially stood in front of the horses’ stall without contacting them. After scoring behavior and visual expression, contact was made with the stallion and the physical examination was done, including recording physiologic data for the modified CPS.

Horses would receive butorphanol as rescue analgesic when the CPS or HGS reached a moderate level of pain. Moderate pain score was defined as one-third of the total score of CPS or HGS. All horses with either a modified CPS >12/36 or HGS >4/12 were eligible for the rescue medication butorphanol (Butorgesic® 10 mg/mL, CP-Pharma, Burgdorf, Germany) 0.1 mg/kg BW i.v.. If a stallion with evidence of pain continued to exceed the limit of CPS or HGS after two consecutive applications of butorphanol, or another condition for pain became clinically apparent, the study had to be discontinued with breaking the blinding for these patients and treatment adapted to the condition was implemented. Horses without postoperative complications were discharged 3 days after surgery. Box rest and hand walking were advised for the following 2 weeks (20–60 min/day). The horses were then allowed to return to their normal level of activity.

### Blood collection and preparation

Twenty ml of blood was collected from the indwelling vein cannula in the jugular vein the day before surgery, 24 h, 48 h, and 72 h after surgery. Before blood sampling, 10 mL of blood were discarded. Blood was stored in EDTA plasma tubes (K3 EDTA, Sarstedt, Nuembrecht, Germany) for hematology, heparin plasma tubes (lithium-heparin, Sarstedt, Nuembrecht, Germany) for clinical chemical examination, cortisol and IL1ß measurements, citrate plasma tubes (Citrate 3,2%, Sarstedt, Nuembrecht, Germany) for fibrinogen measurement and serum was collected in tubes with a clotting activator (serum tube, Sarstedt, Nuembrecht, Germany) for SAA determination. For storage, 3 mL of serum and 2 mL of heparin plasma were obtained at each time point by centrifugation and frozen at −80°C.

### Laboratory

Complete hematology was done with the ADVIA 2120 (Siemens Healthcare GmbH, Erlangen, Germany) or the ProCyte Dx™ (IDEXX, Westbrook, United States). Both analyzers have been validated previously for equine specimen and showed excellent agreement (37).

Clinical chemistry testing with the ABX Pentra C400 (Horiba Diagnostics, France) included urea, creatinine, sodium, chloride, potassium, calcium, phosphate, magnesium, total protein, albumin, globulin, total bilirubin, alkaline phosphatase (AP), glutamatdehydrogenase (GLDH), gamma-glutamyl transpeptidase (GGT), aspartate aminotransferase (AST), creatine kinase (CK), lactate dehydrogenase (LDH), and iron.

Plasma fibrinogen was measured on the STA Compact Max3 (Diagnostica Stago S.A.S., France) and serum SAA concentrations were determined with the immunoturbidometric method (LZ test SAA, Eiken Chemical, Tokyo, Japan) run on the Pentra C400 analyzer.

Cortisol and IL1ß were analyzed in an external laboratory (Laboklin, Bad Kissingen, Germany). Samples were shipped frozen. Cortisol was measured in duplicates with a chemiluminescence assay, validated for equine samples, on an Immulite 2000 XPi System (Siemens Healthcare GmbH, Erlangen, Germany). Equine Interleukin 1ß was measured in duplicates with a sandwich enzyme immunoassay specific for equine samples according to manufacturer’s protocol and absorbance was measured with a 96-plate reader at 450 nm wavelength (Reddot Biotech, Kelowna, Canada).

### Statistics

The standard statistical methods were employed using the JMP software (JMP Statistical Discovery LLC, Cary, United States). Before analysis, the data set was checked for outliers and for normal distribution. The number of stallions per group was calculated to achieve a power of 0.8 calculated for the main variable serum SAA assuming a reduction of serum SAA by Traumeel LT ad us. vet. and Flunixin-Meglumin compared to a previous study (13). The calculated number was 25 stallions per group. To correct for potentially unmatchable horses the study aimed for 60 horses. A retrospective power analysis was also performed showing that, for a reduction of the main variable serum SAA, a power of >0.8 was reached. Baseline data were calculated for the ITT Intention-to-treat population (*n* = 57). All further statistical evaluation was done with the per-protocol-population (PP) (*n* = 49; TR group: *n* = 24; FL group: *n* = 25). Baseline data were compared between groups using the Wilcoxon signed-rank test for interval data. The Wilcoxon signed-rank test was used to test matched-pairs data for a common median, assuming that the population is symmetric. Mantel–Haenszel test for ordinal data was used, when appropriate. For the comparison between treatments, changes from baseline and differences between the groups were calculated. Statistical comparisons were conducted with repeated measures ANOVA to analyze the differences among means, and the Mantel–Haenszel test was used for stratified or matched categorial data. The non-parametric McNemar test was used to analyze paired nominal data. For dependent variables, the paired t-test was used. For all comparisons, two-sided 95% confidence limits were calculated.

A *p*-value <0.05 was considered statistically significant. For nearly normal distributed variables, the data description was given by arithmetic mean and standard deviation. Graphical representation of the data was given by box-and-whisker-plots showing the first (25%), the median (50%) and the third quartile (75%). The whiskers display values within 1.5 times the interquartile range (IQR). Outliers are shown separately and are plotted as individual points.

## Results

**Figure 1:**
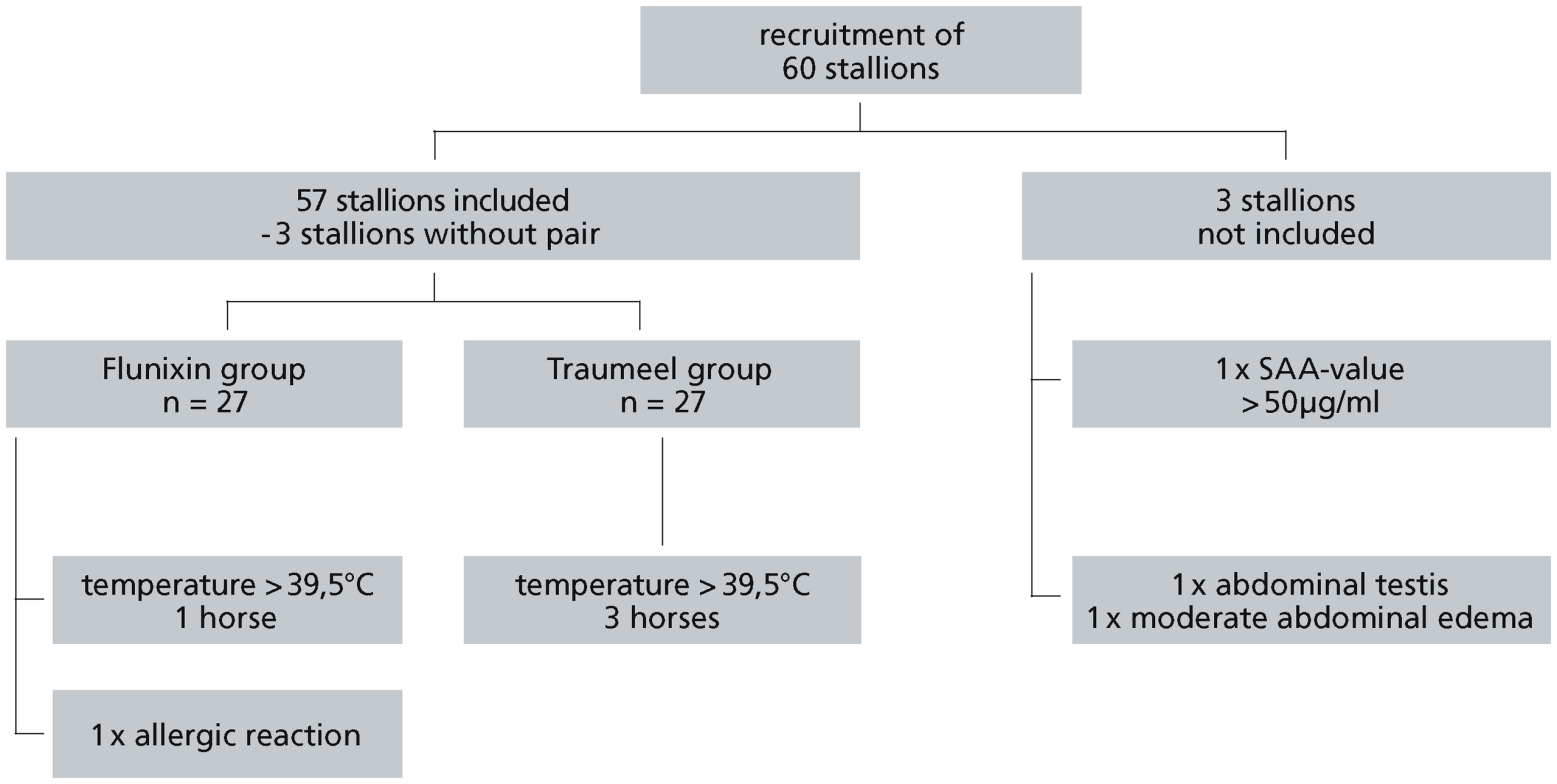
Recruitment of stallions.

Sixty stallions were recruited for the study, 57 stallions fulfilled all inclusion criteria and had no exclusion criteria. Of these 57 stallions 3 stallions had no match in the matched pairs randomization and had to be excluded from the analysis, leaving 54 stallions in the study with 27 stallions in the FL group and 27 stallions in the TR group (Figure 1). All included stallions (*n* = 54/60, ITT) had a mean body weight of 467 +/− 120.3 kg (SD) and a mean age of 3.5 +/− 1.8 years (SD). Descriptive statistics of the results of the initial examination are summarized in Table 1. There was no statistically significant difference between groups for these parameters (Table 1).

**Table 1:** Summary of clinical baseline data (*n* = 54).

Characteristic	TR group (*n* = 27)	FL group (*n* = 27)	*p*- value
Age (years) mean ± SD	3.4 ± 1.6	3.7 ± 2.1	0.49
Weight (kg) mean ± SD	469 ± 116	461 ± 118	0.82
BMI mean ± SD	184.2 ± 26	189.8 ± 21	0.4
Serum SAA (μg/ml) mean ± SD	5.82 ± 1.0	5.77 ± 1.1	0.97
WBC (G/L) mean ± SD	7.65 ± 1.53	7.99 ± 1.76	0.45
Neutrophils (G/L) mean ± SD	4.1 ± 1.06	4.1 ± 1.18	0.93
RBC (´1,012/L) mean ± SD	8.64 ± 0.69	8.36 ± 1.00	0.24
HCT (L/L) mean ± SD	0.38 ± 0.04	0.37 ± 0.04	0.62
Iron (μg/dl) mean ± SD	22.2 ± 7.26	24.8 ± 6.4	0.17
Fibrinogen (g/L) mean ± SD	2.1 ± 0.6	2.1 ± 0.4	0.82
Creatinine (μmol/l) mean ± SD	113.4 ± 16.34	120.7 ± 17.46	0.12
Plasma cortisol (μg/l) mean ± SD	35.09 ± 10.81	34.85 ± 11.88	0.37
Plasma IL1ß (pg/ml) mean ± SD	8.31 ± 12.68	19.08 ± 59.31	0.36

TR, Traumeel LT ad us. vet.; FL, Flunixin-Meglumin; SD, standard deviation; BMI, Body Mass Index; RBC, Red blood cells; HCT, hematocrit; WBC, white bloods cells; IL1ß, Interleukin 1ß.

Of the 57 stallions enrolled in the study, five horses had to be excluded during the study. One of the five horses had an allergic reaction after receiving the α2-agonist and Flunixin-Meglumin for premedication and received a glucocorticoid (dexamethasone 0.08 mg/kg BW i.v., Dexamethasone-injection solution 2 mg/mL, CP-Pharma, Burgdorf, Germany). Four other stallions were noticed with fever above 39.5°C 36 h after castration and were further examined with ultrasound of the abdomen and treated with antibiotics and other NSAIDs. Of these five horses excluded during the study period, two were in the FL group and three in the TR group. This difference was not statistically significant (*p* = 0.46). For further statistical analysis the PP population (*n* = 49) was used.

**Figure 2:**
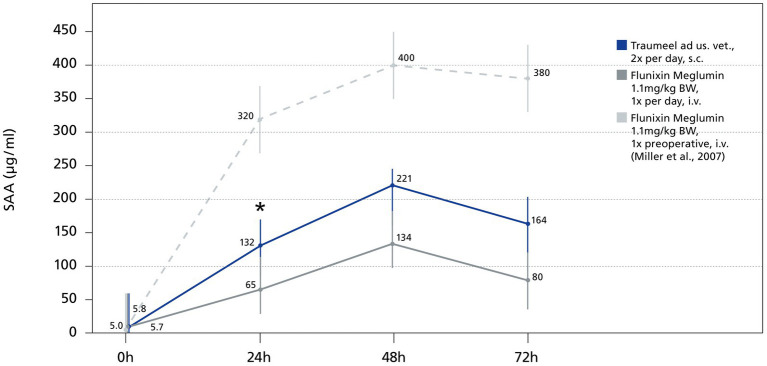
Serum SAA in stallions before and after castration. Mean values of SAA at baseline and at 24 h, 48 h and 72 h are displayed. Dashed, light gray line displays the SAA values of a previous study from Miller et al. (13) with a comparable castration technique, but Flunixin-Meglumin was only given once before surgery. Horizontal lines represent standard error. * Indicates significant difference between groups (*p* < 0.05). Traumeel group (*n* = 24), Flunixin group (*n* = 25).

**Figure 3:**
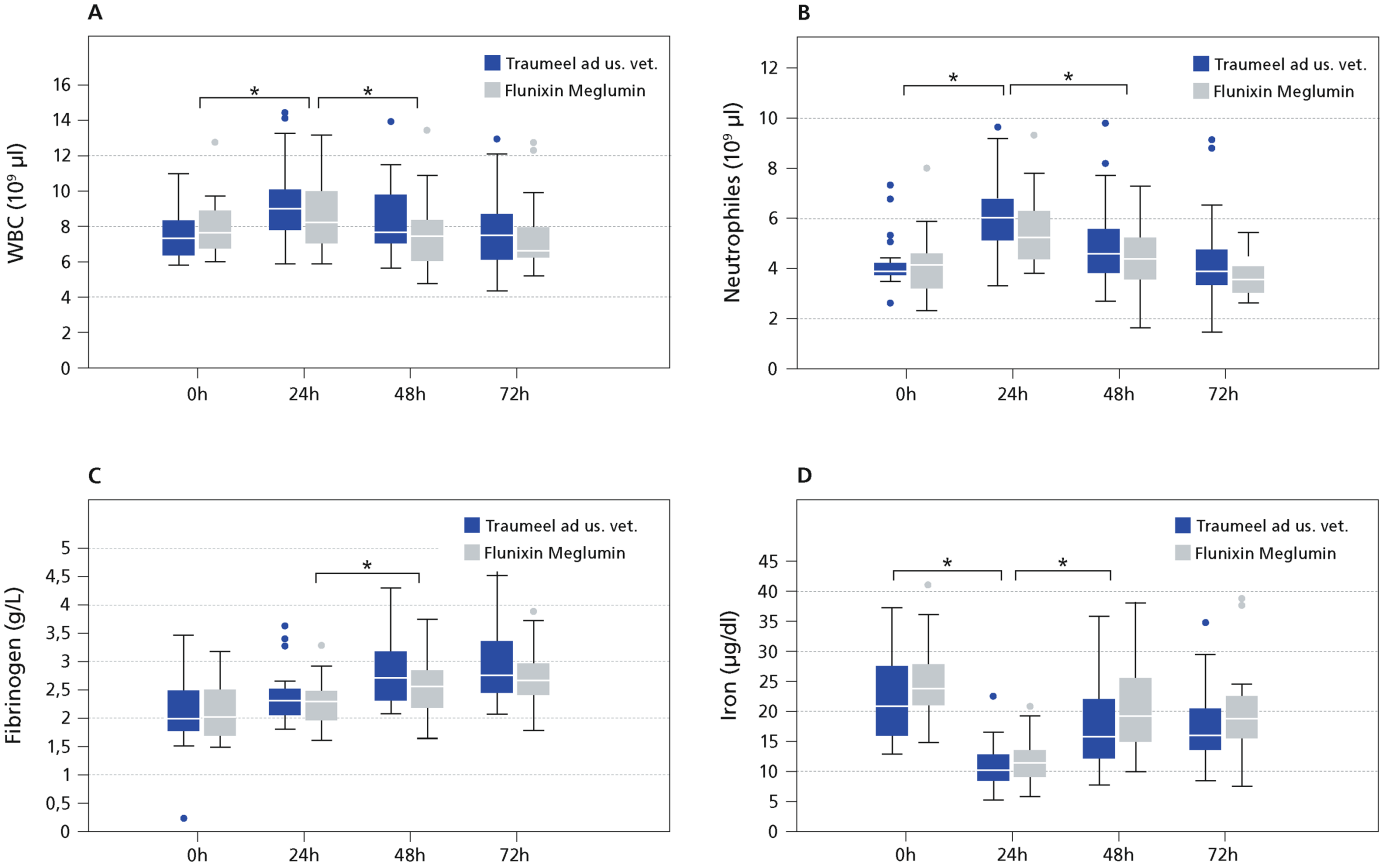
Moderate and Minor APR. The data (*n* = 54) displayed as boxplots showing the first (25%), the median (50%) and the third quartile (75%). The whiskers display values within 1.5 times the interquartile range (IQR). Outliers are shown separately and are plotted as individual points. * Indicates significant difference between groups (*p* < 0.05). Traumeel group (*n* = 24), Flunixin group (*n* = 25). Moderate **(A,B,D)** and minor APR **(C)**.

The major acute phase protein SAA increased significantly within 24 h and 48 h in both groups and decreased at the 72 h timepoint (24 h: TR: *p* = 0.0001, FL: *p* = 0.0012; 48 h: TR: *p* = 0.006, FL: *p* = 0.0092; 72 h: TR: *p* = 0.006, FL: *p* = 0.0002; Figure 2). At the 24 h time point SAA values differed significantly comparing the FL and the TR group (*p* = 0.03). There was no significant difference between the groups at any other time point (0 h: *p* = 0.80, 48 h: *p* = 0.08 72 h: *p* = 0.09). The highest SAA mean value was reached at 48 h (TR: 226 μg/mL, FL: 122 μg/mL). SAA decreased thereafter (Figure 2). Following surgery, the moderate APRs WBC and neutrophils showed an increase induced by the surgery and going back to baseline within 72 h (Figures 3A,B). There was no significant difference between the TR and FL group. Fibrinogen increased mildly but significantly at 24 h and 48 h, going from a baseline value of 2.1 to 2.8 g/L at 72 h not exceeding the reference range. There was no significant difference between both groups at any time point (0 h: *p* = 0.97, 24 h: *p* = 0.23, 48 h: *p* = 0.11, 72 h: *p* = 0.17; Figure 3C). As part of the inflammatory reaction, iron decreased significantly within 24 h after surgery from 23.5 μg/dL at baseline to 11.9 μg/dL at 24 h (*p* < 0.001; Figure 3D). Both groups did not differ significantly at any time point (0 h: *p* = 0.11, 24 h: *p* = 0.25; 48 h: *p* = 0.29; 72 h: *p* = 0.34).

**Figure 4:**
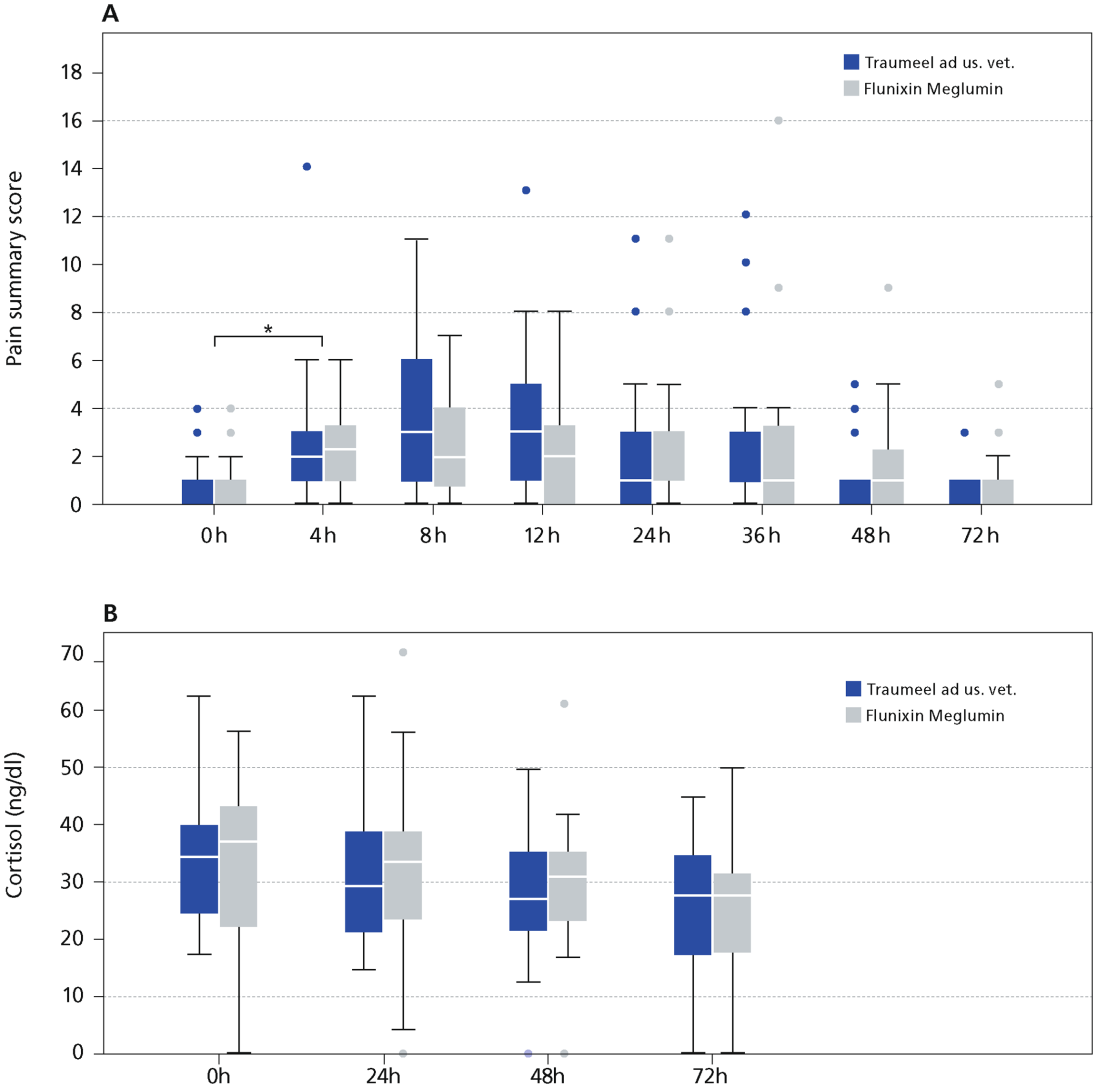
Pain evaluation in Stallions after castration. **(A)** Pain summary score. **(B)** Plasma Cortisol. The data (*n* = 54) displayed as boxplots showing the first (25%), the median (50%) and the third quartile (75%). The whiskers display values within 1.5 times the interquartile range (IQR). Outliers are shown separately and are plotted as individual points. * Indicates significant difference between groups (*p* < 0.05). Traumeel group (*n* = 24), Flunixin group (*n* = 25).

Despite measures of pain reduction, surgery caused a significantly increased pain score in both groups reaching highest values at 8 h post-surgery going back to baseline values at 48 h in the TR group and 72 h in the FL group (Figure 4). There was no significant difference at any time point between the treatment groups for HGS. A *p*-value could not be calculated for the 0 h and 72 h timepoint because the mean of both groups was zero (4 h: TR: *p* = 0.31, 8 h: *p* = 0.8, 12 h: *p* = 0.21, 24 h: *p* = 0.19, 36 h: *p* = 0.16, 48 h: *p* = 1.0). In addition, no statistically significant differences in the modified CPS comparing both groups were seen (before surgery: *p* = 0.42, 4 h: p = 0.2, 8 h: *p* = 0.13, 12 h: *p* = 0.41, 24 h: *p* = 0.34, 36 h: *p* = 0.76, 48 h: *p* = 0.29, 72 h *p* = 0.63). For better visibility a pain summary score was calculated by adding both pain scores (Figure 4). In accordance with the single pain scores, there was no significant difference at any time point in the pain summary score comparing the TR and FL group (before surgery: *p* = 0.37, 4 h: *p* = 0.79, 8 h: *p* = 0.06, 12 h: *p* = 0.1, 24 h: *p* = 0.63, 36 h: *p* = 0.94, 48 h: *p* = 0.4, 72 h *p* = 0.63). Interestingly, there was a fair to good correlation (r^2^: 0.56–0.83) of both scores at all time points except the 0 h and the 72 h time point. No horse included in the study needed the rescue medication butorphanol at any time point. IL 1ß, a pain mediator, was slightly increased not reaching significance. There was no significant difference in the TR and the FL group, comparing the mean IL 1ß concentration (0 h: *p* = 0.39, 24 h: *p* = 0.41, 48 h: *p* = 0.34, 72 h: *p* = 0.33). One horse in the FL group showed high values of IL 1ß before and during the study. Therefore, IL 1ß was also calculated, excluding the outlier from the analysis and no significant difference was seen comparing both groups (0 h: *p* = 0.77, 24 h: *p* = 0.70, 48 h: *p* = 0.87, 72 h: *p* = 0.90). Cortisol is a validated marker for pain in horses. There was no significant difference of plasma Cortisol comparing the TR and the FL group at any time point (0 h: *p* = 0.75, 24 h: *p* = 0.05, 48 h: *p* = 0.42, 72 h: *p* = 0.64; Figure 4).

Anesthesia has an impact on general perfusion and tissue oxygenation, and this can influence tissue healing (38, 39). The mean duration of anesthesia was 50 min (range: 35–70 min) and there was no statistically significant difference of the anesthesia length in both groups (TR: mean = 52 min, FL: mean = 49 min, *p* = 0.23). Xylazine was given as continuous drip infusion as part of the balanced anesthesia protocol. The total xylazine dose was not statistically different in both groups (xylazine total dose: TR: mean = 300.17 mg per anesthesia, FL: mean = 271.06 mg per anesthesia, *p* = 0.27), also the xylazine dose per body weight and time was not significantly different between groups (TR: mean = 0.75 mg/kg per hour, FL: 0.73 mg/kg per hour, *p* = 0.53). During anesthesia the horses received dobutamine at a low dose to maintain a MAP between 60 and 120 mmHg. Dobutamine was given at a group of 0.2–1 μg/kg per min (mean: TR group: 0.49 μg/kg per min, FL 0.57 μg/kg per min; *p* = 0.34). The mean MAP in the stallions was between MAPmin of 60 mmHg and MAPmax of 118 mmHg (mean MAPmin: TR: 80.2 mmHg; FL: 77.5 mmHg, *p* = 0.40 and mean MAPmax: TR: 94.1 mmHg; FL: 95.3 mmHg, *p* = 0.74).

**Figure 5:**
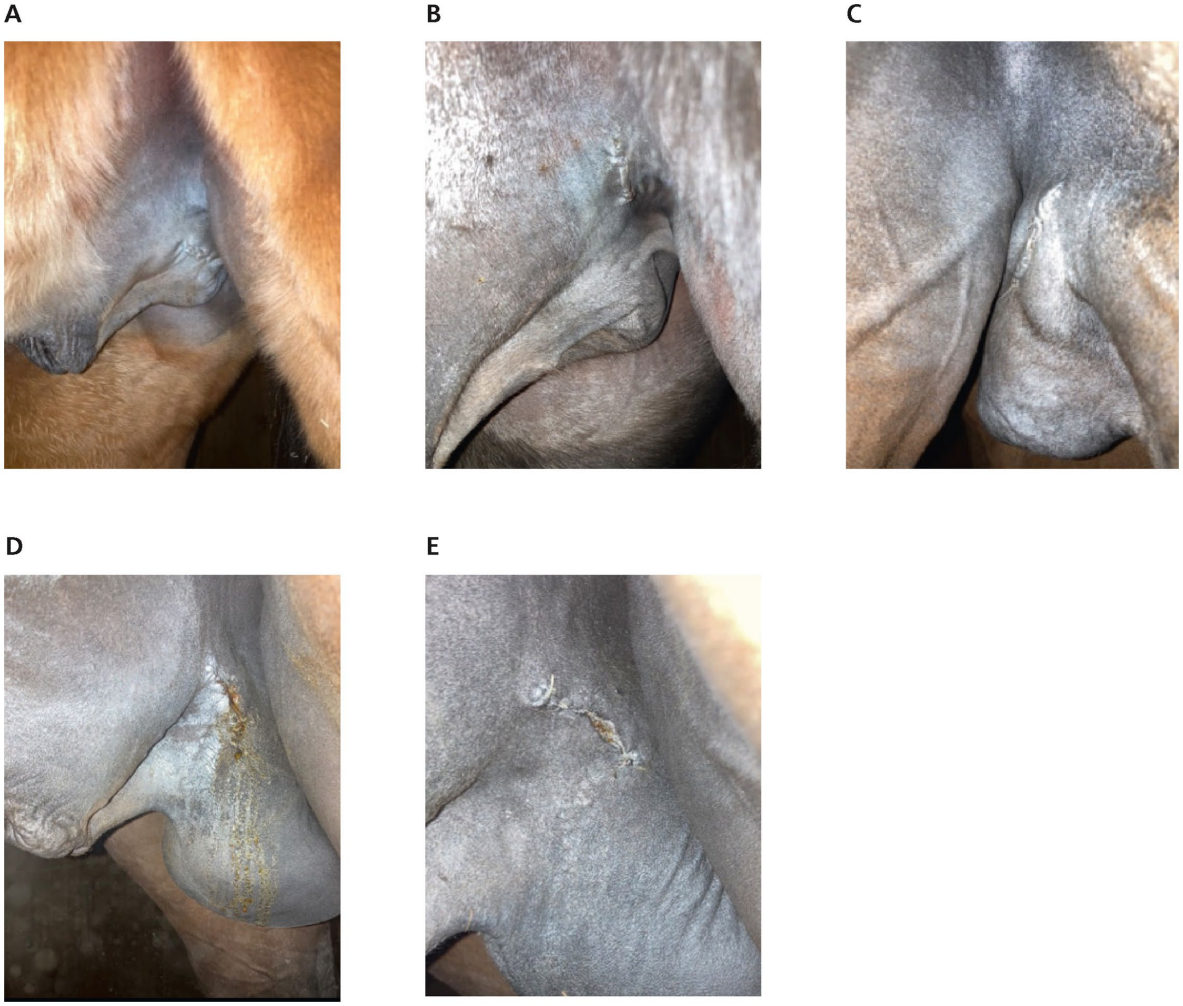
Wound healing. **(A,B)** The suture of the left inguinal space without any signs of wound healing complications, 24 h after castration. **(A)** Horse treated with Traumeel LT ad us. vet. **(B)** Horse treated with Flunixin-Meglumin. **(C)** Mild oedema of the suture (stallion treated with Flunixin-Meglumin) 24 h after castration. **(D,E)** suture dehiscence in a stallion treated with Flunixin-Meglumin. **(D)** 24 h after castration and E. 72 h after castration.

Overall, 12.24% (*n* = 6/49) of stallions had a suture dehiscence of varying degrees. Suture dehiscence occurred 8 h after surgery in one stallion, and 24 h or 36 h after surgery in two and three stallions, respectively. Suture dehiscence was scored as mild in 4/6 stallions and moderate in two stallions. In the FL group 5 stallions developed a suture dehiscence compared to only one stallion in the TR group (*p* = 0.001; Figure 5).

**Figure 6:**
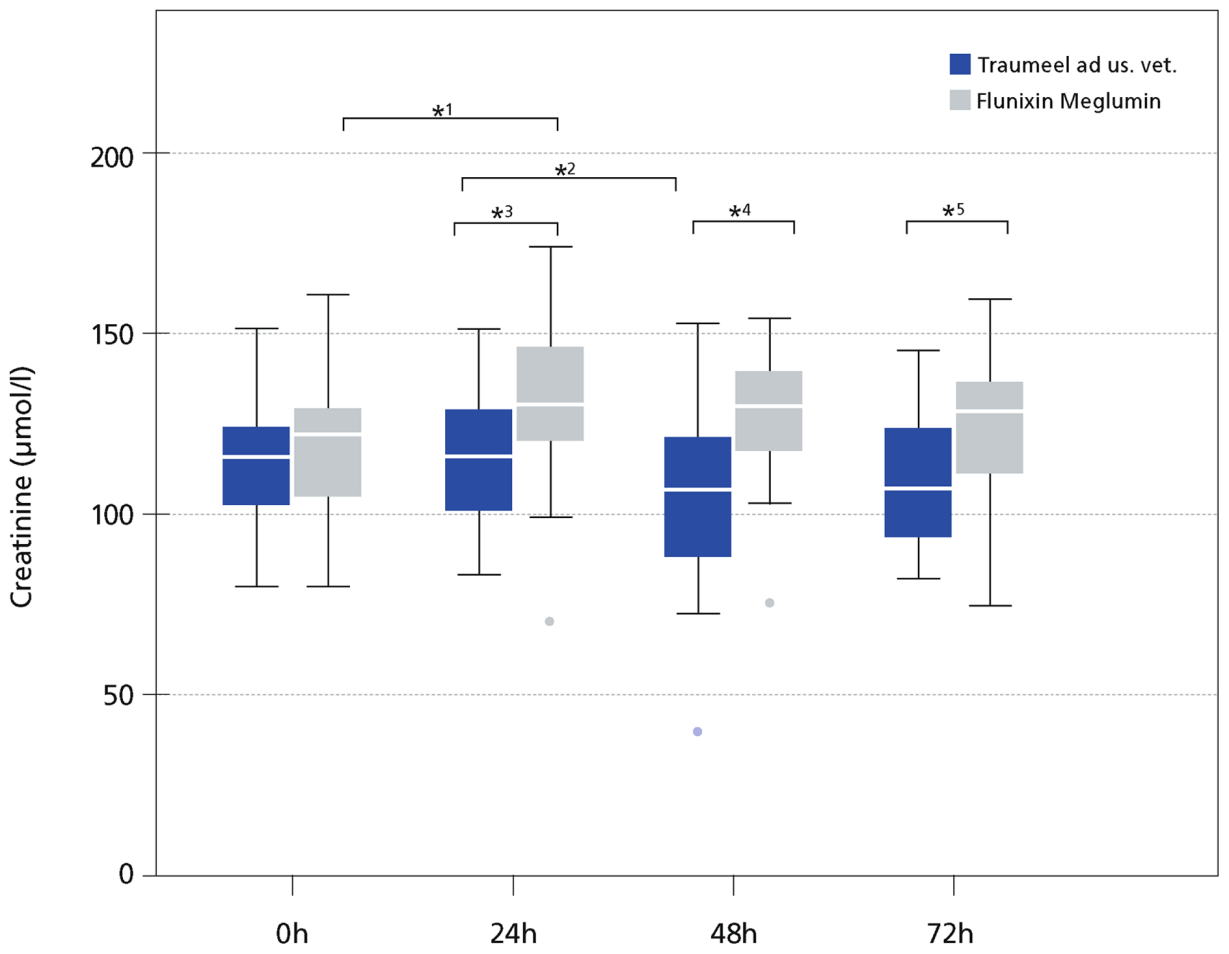
Plasma Creatinine (μmol/l). The data (*n* = 49) displayed as boxplots showing the first (25%), the median (50%) and the third quartile (75%). The whiskers display values within 1.5 times the interquartile range (IQR). Outliers are shown separately and are plotted as individual points. * Indicates significant difference between groups (*p* < 0.05). Traumeel group (*n* = 24), Flunixin group (*n* = 25)1: *p* < 0.0001, 2: *p* = 0.016, 3: *p* = 0.019, 4: *p* = 0.0002, 5: *p* = 0.0049.

In the FL group, creatinine increased after surgery and was significantly higher compared to the TR group at all time points after surgery (0 h: *p* = 0.14, 24 h: *p* = 0.019, 48 h: *p* = 0.0002, 72 h: *p* = 0.0049; Figure 6). In 2/27 horses of the FL group, the creatinine plasma concentration slightly exceeded the upper reference interval 48 h after surgery (162–173.6 μmoL/L, reference interval: 77–160 μmoL/L). There was no correlation of creatinine, and age in this study (*r*^2^ = 0.001; *p* = 0.8; Figure 6).

### Side effects

Traumeel LT ad us. vet. was well tolerated by all stallions. The local reactions at the subcutaneous injection sites were monitored and 4/27 stallions of the FL group receiving NaCl s.c. as placebo and 2/27 stallions from the TR group developed a slight swelling that resolved within a day after injection. No side effects caused by the indwelling jugular catheter were recorded.

## Discussion

The study showed a reduced APR in both treatment groups after castration when compared to stallions receiving Flunixin-Meglumin once before surgery (13). The APR after surgery has been of great interest in the last two decades in equine medicine and surgery, showing a prognostic impact for post operative complications (6, 13, 40) The APR can be characterized by body temperature, WBC, neutrophils, fibrinogen, iron, and SAA. SAA is the most important APP with a short half-life of 30–120 min explaining a fast increase with a drop of SAA within 12 h if inflammation is resolved, making it a good monitor of treatment response (20, 41).

In our study, the tissue damage caused by castration induced a measurable inflammatory reaction in both treatment groups. Previous studies with comparable castration techniques and SAA assay measured peak mean SAA values up to 400–600 μg/mL at 72 h (6, 13). The amplitude and the duration of the SAA increase in our study was lower with peak mean SAA values up to 176.4 μg/mL at 48 h compared to previous studies (6, 13). The increase of SAA is certainly dependent on the castration technique used with standing castration in the stable showing highest values (16) and closed castration in a clinical environment having lowest SAA concentrations (13). Taking the data from comparable studies the duration and amplitude of the SAA concentration in this study seems reduced. For example, the study from Miller et al. used the same castration technique and SAA assay, but Flunixin-Meglumin and antibiotics were administered only once before surgery. In our study, Flunixin-Meglumin was given three times and no antibiotic was administered. The differing dosing regimen can explain the reduced APR in the here presented study.

SAA is not only influenced by the castration technique. Antibiotics given preoperatively can lower SAA by reducing the inflammatory reaction (16) and consequently lowering the risk of complications (42). Reducing the usage of antibiotics, especially in a prophylactic setting before surgery is of foremost importance to reduce the formation of multidrug resistant bacteria (43).

In our study, increased temperature, as one complication, was seen in 7.4% of stallions. Feverish temperature has been assessed in a previous study, reaching 2.5% in stallions with primary wound closure receiving penicillin preoperatively (44). These results show that antibiotics can be avoided in a clinical setting with sterile surgical preparation, close monitoring of the horses and if Traumeel LT ad us. vet. or Flunixin-Meglumin are given pre-and postoperatively to avoid complications by reducing the APR after surgery.

Flunixin-Meglumin is a well-known potent anti-inflammatory drug reducing SAA by inhibiting COX-1 and COX-2 and thereby reducing the APR after stallion castration (25). The strongest effect of Flunixin-Meglumin on SAA after surgery has been seen if Flunixin-Meglumin is given i.v. before and over 48-72 h post operatively (25). The effect was less pronounced if Flunixin-Meglumin was only given once before surgery (13) and this might explain comparatively reduced activation of the APR seen in this study.

**Figure 7:**
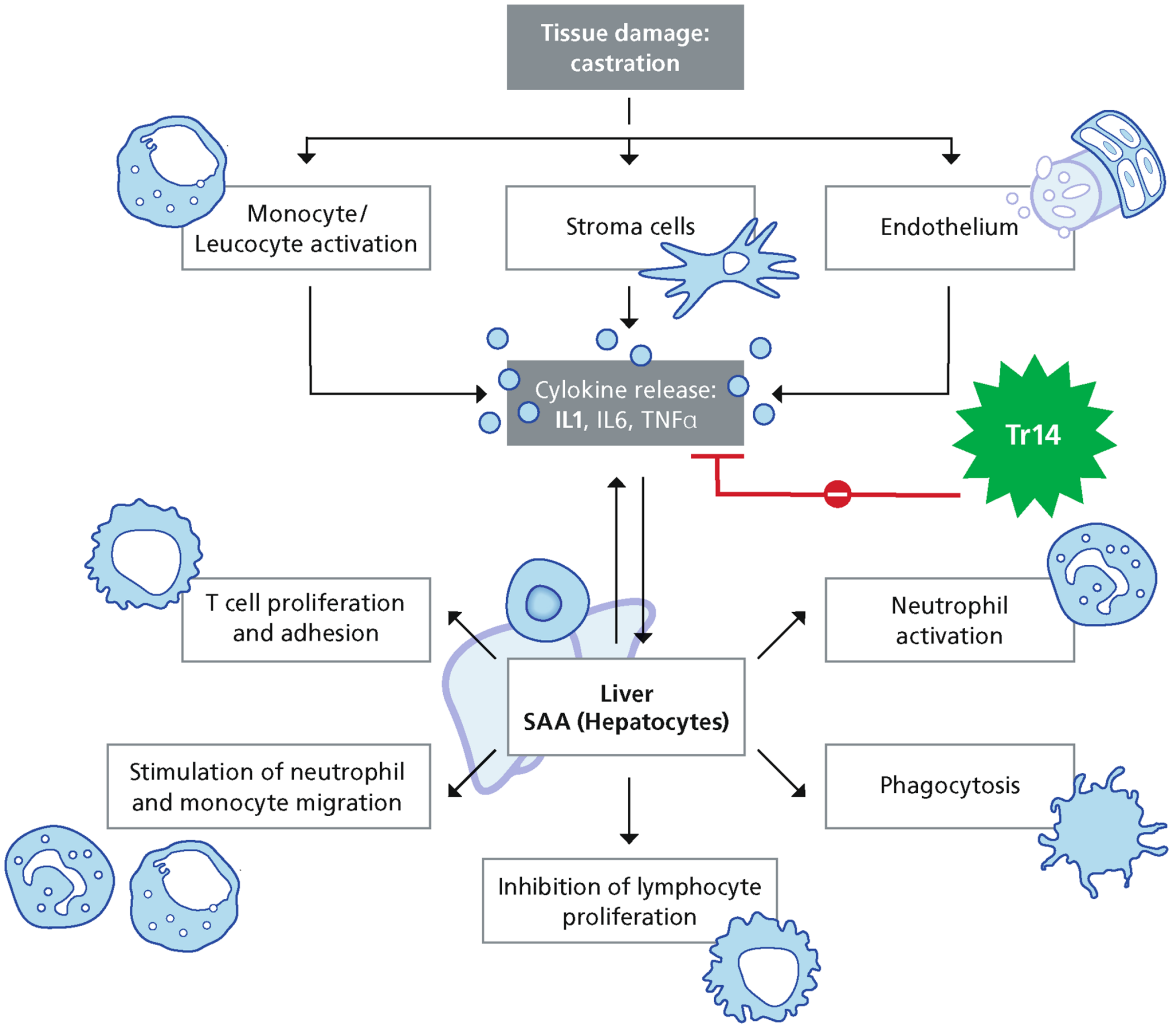
Pathways involved in SAA increase after surgery.

Traumeel is known to actively induce inflammation resolution via down regulation of IL1ß, and specific proresolving mediators (SPMs) (31). Traumeel is also shifting the pro-inflammation cytokines prostaglandin and leucotriene toward the pro-resolving mediator’s prostacyclin and lipoxin, thereby inducing the switch of pro-inflammatory macrophages to efferocytic macrophages. This mode of action of Traumeel might explain the reduced APR seen in this study (Figure 7). The significant difference at the 24 h timepoint can be explained by the differing mode of action of Traumeel and Flunixin-Meglumin. After resolution of inflammation, tissue repair can be initiated allowing a faster onset of wound healing (31). Traumeel has also a positive impact on wound healing by regulating over 100 mRNA transcripts related to key wound repair pathways, such as response to wounding, wound contraction, and cytokine response in a mouse model (2, 30), this mode of action might explain the reduced number of stallions with suture dehiscence in the TR group compared to the FL group (Figure 7).

In a previous study, Traumeel reduced the pro-inflammatory cytokine IL1ß significantly. IL1ß is considered to be responsible for inflammation induced pain (2), also Flunixin-Meglumin can inhibit pro-inflammatory cytokines such as IL1ß efficiently (45), in concordance to these previous findings Traumeel LT ad us. vet. and Flunixin-Meglumin seemed to down regulate IL1ß efficiently.

In concordance to the reduced SAA values, Traumeel LT ad us. vet. seemed to also have an influence on WBC, neutrophils, fibrinogen, and iron. For example, fibrinogen demonstrates a more gradual and persistent response compared to SAA with at least doubled values after castration. Fibrinogen did not exceed the normal range and values did not even double within that time in our study as seen in other studies (6, 8). Also, the amplitude and the duration of all other measured minor and moderate inflammatory parameters were indicative for a mild APR after castration in this study.

Every surgical procedure causing a disruption of local tissue homeostasis is causing pain. Multiple measures have been taken to reduce pain in this study. First, the castration technique used, second the local anesthetic injected in both testis and spermatic cords and third the treatment with either Flunixin-Meglumin or Traumeel LT ad us. vet.. The Composite Pain Scale and the Horse Grimace Scale are validated, reliable and reproducible pain scales (23, 24, 36). The pain scores only reached values indicative for mild to moderate pain, and butorphanol as rescue medication was not necessary at any time point. At the first time point (4 h) after surgery pain could have been influenced by Xylazin, a potent pain killer. The dose of Xylazine was the same in both groups, assuming that the effect was similar in both groups. The subjective pain assessment was further supported by plasma cortisol measurements. Cortisol has been evaluated in horses as a good marker for postoperative pain (46, 47). Cortisol increased just before surgery with declining values within the observation time of 72 h. Cortisol is also a well-known marker for induction of the stress axis by stressors such as transport and handling (48–50). The increase of cortisol before surgery might be stress induced by transport and handling. After a surgical procedure, the stress axis can be activated by pain causing an increase in plasma cortisol. There was no statistically significant difference comparing the FL and the TR group at any time point for the HGS, the modified CPS and plasma cortisol, indicating that the pain was well controlled in this study in either treatment arm. In agreement with our results, Flunixin-Meglumin has been shown to efficiently reduce the APR response after castration and to efficiently control pain in horses (28, 51–54).

In horses, the gastrointestinal (GI) tract and kidneys are considered the organs most affected by the side effects of NSAIDs (29, 55). The mechanism of action of NSAIDs involves the inhibition of COX1/COX2. These enzymes are responsible for synthesizing prostanoids, which are important to prevent GI tract disease and regulate renal blood flow to correct hypovolemia (53, 56, 57). In our study, stallions with known or suspected GI tract disease or known renal disease were excluded to avoid complications. Nevertheless, in the FL group, creatinine was significantly increased 24 h after surgery and was significantly increased compared to the TR group showing the different mode of actions of both treatment arms.

The main limitations of the study were first that no placebo group was included due to animal welfare reasons. Second, the anesthesia protocol controlled for MAP but was not controlled for xylazine and dobutamine and that might have influenced tissue perfusion (38). Also, a longer observation period would have been of interest to monitor the time from the upregulation of SAA to reach baseline values and to assess the overall time necessary for wound healing. In this clinical setting, the stallions had to be released from the clinic after 72 h post-surgery, making a longer observation time infeasible. Measuring SPMs in the wounded tissue would have been interesting, but taking biopsies in the wounded area would have limited the number of owners willing to participate in the study.

Within the limitations of this study, Traumeel LT ad us. vet. seems to have proresolving effects after stallion castration making it a valuable treatment to reduce the APR induced by castration, and thereby reducing postsurgical complications. Due to its different mode of action Traumeel LT ad us. vet. might be an alternative treatment option if gastrointestinal side effects or renal side effects of NSAIDs should be avoided. A further study is needed to clarify if a combination of Traumeel LT ad us. vet. and Flunixin-Meglumin preoperatively, continuing with Traumeel LT ad us. vet. after surgery would have beneficial effects compared to each treatment group alone.

## Data Availability

The original contributions presented in the study are included in the article/Supplementary material, further inquiries can be directed to the corresponding author/s.
