# Supplementary material for: The effect of Traumeel LT ad us. vet. on the perioperative inflammatory response after castration of stallions: a prospective, randomized, double-blinded study
Source: Front Vet Sci. 2024 Oct 2;11:1342345. doi: 10.3389/fvets.2024.1342345 (PMC11480072; doi:10.3389/fvets.2024.1342345)
Supplement: Supplementary file 1 [file Table_1.docx]

Supplemental Table 1

| Hematology | |
| --- | --- |
| White blood cells | 3.5-9.4 x 10^9^/l |
| Neutrophils | 1.6-6.4 x 10^9^/l |
| Lymphocytes | 1.2-4.0 x 10^9^/l |
| Monocytes | <0.6 x 10^9^/l |
| Eosinophils | <0.7 x 10^9^/l |
| Basophils | <0.30 x 10^9^/l |
| Red blood cells | 6.2-8.9 x 10^12^/l |
| Hemoglobin | 6.30-9.0 mmol/l |
| Hematocrit | 0.30-0.43 l/l |
| Reticulocytes | 1.79-7.79 x 10^9^/l |
| Mean corpuscular volume | 42-55fl |
| Mean corpuscular hemoglobin concentration | 20.38-22.22 mmol/l |
| Platelets | 80-230 x 10^9^/l |
| Clinical chemistry | |
| Urea | 3.0-7.1 mmol/l |
| Creatinine | 77-160 μmol/l |
| Sodium ionized | 133-144 mmol/l |
| Chloride | 92-102 mmol/l |
| Potassium ionized | 2.6-4.4 mmol/l |
| Calcium | 1.50-1.80 mmol/l |
| Total Protein | 57.7-72.9 g/l |
| Albumin | 27.4-35.7 g/l |
| Globulin | 24.7-41.5 g/l |
| Bilirubin | 11.1-51.8 μmol/l |
| Alkaline phosphatase | <260 U/l |
| Glutamate-dehydrogenase | <145 U/l |
| Gamma glutamyl transferase | <140 U/l |
| Aspartate- aminotransferase | <800 U/l |
| Creatine kinase | <450 U/l |
| Lactate dehydrogenase | <640 U/l |
