# Supplementary material for: The effect of Traumeel LT ad us. vet. on the perioperative inflammatory response after castration of stallions: a prospective, randomized, double-blinded study
Source: Front Vet Sci. 2024 Oct 2;11:1342345. doi: 10.3389/fvets.2024.1342345 (PMC11480072; doi:10.3389/fvets.2024.1342345)
Supplement: Supplementary file 2 [file Table_2.docx]

Supplemental Table 2:

Supplemental Table 2a: modified Composite Pain Scale (CPS)

|  | Criteria | Score |
| --- | --- | --- |
| Behavior | | |
| Appearance (reluctance to move, restlessness, agitation, and anxiety) | Bright, lowered head and ears, no reluctance to move | 0 |
|  | Bright and alert, occasional head movements, no reluctance to move | 1 |
|  | Restlessness, pricked up ears, abnormal facial expressions, dilated pupils | 2 |
|  | Excited, continuous body movements, abnormal facial expression | 3 |
| Sweating | No obvious signs of sweat | 0 |
|  | Damp to the touch | 1 |
|  | Wet to the touch, beads of sweat are apparent over the horse’s body | 2 |
|  | Excessive sweating, beads of water running off the animal | 3 |
| Kicking at abdomen | Quietly standing, no kicking | 0 |
|  | Occasional kicking at abdomen (1–2 times/5 min) | 1 |
|  | Frequent kicking at abdomen (3–4 times/5 min) | 2 |
|  | Excessive kicking at abdomen (>5 times/5 min), intermittent attempts to 3 lie down and roll | 3 |
| Pawing on the floor (pointing, hanging limbs) | Stands quietly, no pawing | 0 |
|  | Occasional pawing (1–2 times/5 min) | 1 |
|  | Frequent pawing (3–4 times/5 min) | 2 |
|  | Excessive pawing (>5 times/5 min) | 3 |
| Posture (weight distribution, comfort) | Stands quietly, normal walk | 0 |
|  | Occasional weight shift, slight muscle tremors | 1 |
|  | Non-weight bearing, abnormal weight distribution | 2 |
|  | Analgesic posture (attempts to urinate), prostration, muscle tremors) | 3 |
| , Head movement | No evidence of discomfort, head straight ahead for the most part | 0 |
|  | Intermittent head movements laterally or vertically, occasional looking at flanks 1 (1–2 times/5 min), lip curling (1–2 times/5 min) | 1 |
|  | Intermittent and rapid head movements laterally or vertically, frequent looking at flank 2 (3–4 times/5 min), lip curling (3–4 times/5 min) | 2 |
|  | Continuous head movements, excessively looking at flank (>5 times/5 min), lip curling 3 (>5 times/5 min) | 3 |
| Appetite | Eats hay readily or is not allowed to eat hay prior surgery | 0 |
|  | Hesitates to eat hay | 1 |
|  | Shows little interest in hay, eats very little or takes hay in mouth but does not chew or swallow | 2 |
|  | Neither shows interest in nor eats hay | 3 |
| Interactive behavior | | |
| Response to observer | Pays attention to people | 0 |
|  | Exaggerated response to auditory stimulus | 1 |
|  | Excessive-to-aggressive response to auditory stimulus | 2 |
|  | Stupor, prostration, no response to auditory stimulus | 3 |
| Physiologic data | | |
| Rectal temperature (°C) | 36,9 - 38,5 | 0 |
|  | 36,4 - 36,9 or 38,5 - 39,0 | 1 |
|  | 35,9 - 36,4 or 39,0 - 39,5 | 2 |
|  | 35,4 - 35,9 or 39,6 - 40,0 | 3 |
| Heart rate  (beats/minute) | 24 - 44 | 0 |
|  | 45 - 52 | 1 |
|  | 53 - 60 | 2 |
|  | >60 | 3 |
| Respiratory rate  (breaths/minute) | 8 - 13 | 0 |
|  | 14 - 16 | 1 |
|  | 17 - 18 | 2 |
|  | >18 | 3 |
| Digestive sounds (bowel movements) | Normal motility | 0 |
|  | Decreased motility | 1 |
|  | No motility | 2 |
|  | Hypermotility | 3 |
| Total score (maximum) | | 36 |

Supplemental Table 2b: Horse Grimace Scale (HGS)

| Facial Action Unit (FAU) |  | Score |
| --- | --- | --- |
| Stiffely backwards ears | not present | 0 |
|  | moderately present | 1 |
|  | obviously present | 2 |
| Orbital tightening | not present | 0 |
|  | moderately present | 1 |
|  | obviously present | 2 |
| Tension above the eye area | not present | 0 |
|  | moderately present | 1 |
|  | obviously present | 2 |
| Prominent strained chewing muscle | not present | 0 |
|  | moderately present | 1 |
|  | obviously present | 2 |
| Mouth strained and pronounced chin | not present | 0 |
|  | moderately present | 1 |
|  | obviously present | 2 |
| Strained nostrils and flattening of the profile | not present | 0 |
|  | moderately present | 1 |
|  | obviously present | 2 |
| Total score (maximum) | | 12 |
