# Supplementary figures and images for: The effect of Traumeel LT ad us. vet. on the perioperative inflammatory response after castration of stallions: a prospective, randomized, double-blinded study
Source: Front Vet Sci. 2024 Oct 2;11:1342345. doi: 10.3389/fvets.2024.1342345 (PMC11480072; doi:10.3389/fvets.2024.1342345)

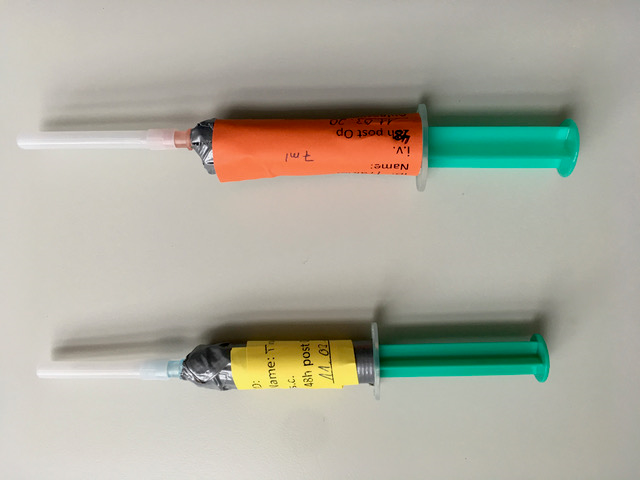

Supplement: Supplementary file 3 [file Image_1.tif]
